# Perceived Barriers to Antepartum HIV Medication Adherence in HIV Infected Pregnant Women

**DOI:** 10.1155/2018/4049212

**Published:** 2018-10-16

**Authors:** Leilah Zahedi-Spung, Marisa Young, Lisa B. Haddad, Martina L. Badell

**Affiliations:** Department of Gynecology and Obstetrics, Emory University School of Medicine, Glenn Building, 4th Floor-412B, 69 Jesse Hill Jr Drive SE, Atlanta, GA 30303, USA

## Abstract

**Introduction:**

Although rare, perinatal HIV transmission still occurs in the United States and most transmissions are preventable. We aim to identify patient barriers to antiretroviral therapy (ART) adherence during pregnancy and assess patient understanding of perinatal transmission.

**Methods:**

This cross-sectional survey recruited HIV positive postpartum women at a large safety net hospital in Atlanta, Georgia, between January 2016 and February 2018. Survey questions included demographic characteristics, HIV history, knowledge of perinatal transmission, and ART adherence. Perinatal and HIV outcomes were assessed using chart abstraction.

**Results:**

Of the 70 HIV infected postpartum women delivered at a large safety net hospital in Atlanta, GA, 45 women were eligible and consented to participate. Participating women were aged 18 to 40 years with an average age of 29 years old, 93% of participants were African-American, and 68% had ≥3 pregnancies. The majority of participants (75%) reported daily ART adherence. “Forgetting” was the most frequent reason for missing pills (57%). Thirteen women had a detectable viral load at the time of delivery and nine of those women had a viral load greater than 1000 copies/mL. Approximately 85% of women who correctly stated ART medications decrease perinatal transmission risk reported daily adherence compared with 50% of women without that knowledge (OR 5.6, 95% CI 1.17, 26.7). Almost half of women (40%) either did not know or believed a vaginal delivery, regardless of viral load, would increase their risk of perinatal transmission.

**Conclusion:**

Overall, women who were diagnosed with HIV during the current pregnancy, those with planned pregnancies, and those who were on medications prior to pregnancy were more likely to report daily ART adherence. Detectable viral load at delivery is the greatest risk factor for perinatal transmission; therefore strategies to increase ART adherence are needed.

## 1. Introduction

Approximately 25% of people living with HIV in the US are women [1–4]. In the early years of HIV, infected women had a 25% risk of perinatal transmission [5, 6]. Advances in antepartum management and ART have led to a dramatic reduction in the perinatal transmission of HIV. Current recommendations have led to a perinatal transmission rate of less than 2% in the United States and Europe combined [6–9].

The Enhanced Perinatal HIV Surveillance Programme in Georgia noted that between 2005 and 2011 the perinatal transmission rate of HIV in Georgia was 2.5% [10]. There is also a significant race disparity with regard to perinatal transmission of HIV in the US, with a transmission rate of 12.3/100,000 among African Americans, compared with 0.5/100,000 in Caucasians. In 2016, it was noted that there were 13, 447 women living with HIV in Georgia. In addition, between 2009 and 2016, 43 infants were perinatally infected and 68% were born in the metro Atlanta area [10]. In 2010, 162 cases of perinatally acquired HIV infections were documented in the US A recent study in Georgia noted that there were missed opportunities to prevent transmission in 74.3% of those cases [6]. The study identified 27 perinatally infected infants and found in 24 of the 27 cases limitations in healthcare delivery and uptake were identified as significant risk factors for perinatal transmission. They also noted that 74% of women knew their HIV status prior to pregnancy but only 50% received prenatal care. This study identified several risk factors for perinatal transmission, including illicit drug use, lack of prenatal care, and lack of ART antepartum [6].

A recent series of systematic reviews identified individual and contextual factors and health system barriers affecting ART initiation, adherence, and retention in HIV infected pregnant women [3, 9, 11–14]. Hodgson et al. identified lower age, lower education level, HIV denial, concern ART will harm the child, misplacing/forgetting medication, use of drugs or alcohol, transportation problems, and negative attitudes of health workers as barriers to ART adherence. Lack of knowledge regarding prevention of perinatal transmission was a barrier to initiation of ART [11]. Colvin et al. identified that models of care where ART and antenatal care services were fully integrated were an enabler to initiation, adherence, and retention of ART. A recurring theme was the loss of focus and prioritization of maternal ART within antenatal care and prevention of perinatal transmission programs. They also reported communication and coordination problems as a barrier to initiation, adherence, and retention of ART. Very few of the studies in these systematic reviews were performed in the US It is important to recognize and study what is happening in our own population of HIV infected women [6, 8, 9, 15, 16]. Our study aims to address maternal perceived barriers to ART adherence antepartum among HIV positive women delivering at a large hospital safety net hospital. Knowing that ART can dramatically reduce the risk of perinatal transmission, opportunities for improvement in ART initiation and adherence antepartum could help to reduce the perinatal transmission rate in Georgia.

## 2. Materials and Methods

This cross-sectional survey study recruited HIV positive postpartum women at a large safety net hospital in Atlanta, GA between January 2016 and February 2018. All HIV infected women who delivered a viable fetus at between the ages of 18 and 45 years old, and who spoke and read English were eligible for enrollment. Key exclusion history included women with an uncontrolled psychiatric illness, unknown or negative HIV status, unable or unwilling to consent to the study, or intrauterine fetal demise or fetal loss <20 weeks.

Once an eligible HIV infected patient delivered, the study personnel were contacted. All eligible postpartum patients were approached by study personnel in their room during their inpatient stay on the postpartum floor. Patients were asked to participate in the study while alone in their postpartum inpatient rooms by study investigators. The participants were asked to complete an anonymous 10-minute survey. The survey, provided in supplementary files (available here), included questions regarding: Demographic characteristics, HIV history, knowledge regarding perinatal transmission, reported ART adherence, perceived barriers to ART adherence, and interpersonal and relationship questions.

Following completion of the survey, a chart review was performed in order to collect information regarding their delivery mode, CD4 count and viral load, and neonatal status. Both the survey results and chart review were stored on an online, secure, and HIPPA-compliant database. The study protocol was approved by the Emory University Institutional Review Board and written informed consent was acquired from all study participants.

Coprimary outcomes were rate of reported ART adherence antepartum and perceived barriers to ART adherence. Secondary outcomes included perinatal transmission knowledge, viral load and CD4 count at delivery, mode of delivery, and neonatal HIV status. Descriptive statistics were generated and categorical variables were compared using chi-square tables to produce odds ratios and 95% confidence intervals.

## 3. Results

During the study period, 70 HIV infected postpartum women delivered a viable fetus at a large safety net hospital in Atlanta, Georgia. 45 women were eligible and consented to participate. Of the other 25 women, 13 were ineligible due to age less than 18 years old or language barrier and 12 declined to participate. Participating women were aged 18 to 40 years with a mean age of 29.7 years old (SD +/- 5.8 years), 93% of participants were African-American, and 68% had ≥3 pregnancies. See Table 1 for demographic information.

Approximately half (22/45) of the women had either spontaneous vaginal deliveries or vaginal births after cesarean deliveries and a third of women (15/45) had repeat cesarean deliveries. 80% of women attended 4 or more prenatal care visits. Seventeen women (38.6%) received intrapartum AZT and 38 women had a CD4 count > 250 cells/uL (median 356 cells/uL, IQR 235 cells/uL) at the time of delivery. There were no cases of perinatal transmission in the study population.

### 3.1. ART Adherence

The majority of participants (75%) reported daily ART adherence. When asked for reasons for missed ART doses, “forgetting” was the most frequently cited reason (57%) by all participants. Ten women reported “other” as their reason for missing the pills. Write in responses included, “being busy,” “at work or asleep,” “I had no insurance,” “rushing for an event and end up forgetting,” and “did not have meds or insurance.” Table 2 further describes reported ART adherence. Of the women who reported daily ART adherence, 20 of those women also reported “forgetting” their medication when asked for a reason for missing medication doses.

Among the six women with congenitally acquired HIV, 4 women reported every day ART use. Thirteen women had a detectable viral load at the time of delivery and nine of those women (20%) had a viral load greater than 1000 copies/mL. Of the 13 women with a detectable viral load, 7 women reported daily ART adherence to medication, compared to the women who did not have a detectable viral load, 27 of 32 women reported daily adherence (OR 0.22, CI 0.05, 0.92).

All 7 women who were diagnosed with HIV during their current pregnancy reported daily ART adherence, compared with 68% of women who were diagnosed when they were not pregnant (p=0.08). Approximately 85% of women who were on ART prior to pregnancy reported daily adherence, compared with 67% of women who were not on ART prior to pregnancy (OR 2.9, CI 0.66, 13.55). Women who had been diagnosed with HIV three or fewer years ago had a lower reported daily adherence (72%) compared with those diagnosed 4-10 years ago (85%), and more than 10 years (83%). All of the women (12/45) who had been infected with HIV for > 10 years had a viral load < 1000 copies/mL at time of delivery.

### 3.2. Perinatal Transmission Knowledge

Approximately 85% of women who correctly stated ART medications decrease perinatal transmission risk reported daily adherence compared with 50% of women without that knowledge (OR 5.6, 95% CI 1.17, 26.7). Only 67% of women with an unplanned pregnancy reported daily adherence, compared with 94% of women with a planned pregnancy (OR 7.5, CI 0.84, 67.3). Most women (77.3%) women correctly responded that if they took their ART throughout pregnancy and had a viral load < 1000 copies/mL the risk of HIV transmission was <2%. When asked whether vaginal delivery, regardless of viral load, would increase their risk of perinatal transmission, 40% of women either did not know or reported that was a true statement.

Approximately 15% of women reported they believed the risk of their child being born with HIV was over 10%, some (n=6) reporting as high as 60%. There were seven women who responded that they believed ART medication will harm themselves or their baby.  Table 3 further outlines the knowledge that participants had of perinatal transmission.

At the conclusion of the survey participants were asked if there was anything else their medical providers could have done to make taking their medications easier or more accessible. A few responses included “No I just took it cause I did not want my baby to get HIV,” “I should have reported that I was becoming nauseous so that proper adjustments could have been made to medication and I could have continued taking them more comfortably,” “arrangements for transportation,” “The pills are very big so it was difficult for me to swallow,” “somebody to come out to home and administer the medications to me.”

## 4. Discussion

One-quarter of HIV infected pregnant women in our study reported nonadherence with daily ART during pregnancy. We found the most common reason for this was “forgetting to take pills”. Women who were diagnosed with HIV during the current pregnancy, those with planned pregnancies, and those who were on medications prior to pregnancy were more likely to report daily ART adherence. The time of HIV diagnosis may represent an opportunity for counseling on family planning and reiterating the importance of medication adherence. Innovative strategies are needed to address the ongoing barriers to adherence (forgetfulness and side effects) among pregnant women. Given that detectable viral load at delivery is the greatest risk factor for transmission, and combined ART has the biggest effect on viral load reduction, an important focus during prenatal care for women with HIV should be on medication adherence. As forgetting is a primary reason for nonadherence, future strategies could employ technology such as phone applications or reminders to assist in medication adherence regimens. Starting in 2011, the CDC created an e-learning training toolkit for clinical and nonclinical providers of HIV infected patients. The “Every Dose Every Day” e-Learning Training Toolkit containing four evidence-based strategies. These include individual and group sessions to discuss social support during initiation of ART, discordance between couples, continuation of adherence, and promotion of the patient-provider relationship. Provided has part of the toolkit is a mobile phone application that sets dose of medication, tracks appointments, lab results, and allows patient and provider to communicate regarding medication adherence [17]. Implementing these interventions may contribute to improved ART adherence in HIV infected pregnant women.

Knowledge regarding perinatal transmission is a potential factor contributing to poor medication adherence. A few women in our cohort believed that ART would harm them or their baby. As providers we should continue to educate our patients regarding the safety of ART while pregnant and assess any apprehension to maintaining adherence with medication. In addition, almost half of study participants had a misconception regarding the safety of vaginal delivery in the setting of maternal HIV. Providers should continue educating patients regarding safety of vaginal delivery from a maternal and fetal stand-point if HIV well-controlled.

The patient population in our study has a higher African-American population than the general HIV infected population in the country (93.3% v. 58% respectively). This is largely due to the fact that the patient population cared for is largely African-American. That being said, African-American women have a significantly higher risk of perinatal transmission of HIV and therefore this maximized our ability to study a high-risk population [1].

The biggest limitation of this project is our small sample size. The study personnel relied on the labor and delivery team to inform them of HIV infected patients who presented for delivery in order to recruit patients. This work flow may have led to patients being missed and therefore falsely lowering our sample size. Additionally, only those women willing to participate in the study were surveyed which may lead to selection bias or social desirability bias. Given survey was anonymous and all questions were optional, we had a number of surveys that were incomplete. With regard to ART adherence, we asked women to think back throughout their pregnancy and decide on a weekly basis how often they took their medication. This made us vulnerable to recall bias. In a future study, we should consider surveys regarding medication adherence at every prenatal visit asking about the adherence for the previous week. Given the limited scope of this study, we did not collect or stratify information regarding ART regimens that participants were taking. The differing regimens certainly may impact adherence and therefore in future studies, we would recommend collecting information regarding ART regimens to determine if there are certain regimens that impact adherence.

Future endeavors should implement an improvement plan for those women who are considered to be high risk for poor ART adherence while following their viral load through delivery to determine if it was successful. Providers should continue educating patients during pregnancy regarding the safety of ART and vaginal delivery with a controlled viral load.

## 5. Conclusions

One-quarter of women were not compliant with daily ART during pregnancy. Overall, women who were diagnosed with HIV during the current pregnancy, those with planned pregnancies, those who were on medications prior to pregnancy, and those who correctly understood reduction in perinatal transmission with ART were more likely to report daily ART adherence. There is a persistent misunderstanding regarding risk of vaginal delivery in the setting of maternal HIV. Detectable viral load at delivery is the greatest risk factor for transmission; strategies to address ART adherence are needed.

## Figures and Tables

**Table 1:** Demographics of cohort of HIV infected pregnant women.

	N=45 (%)
*Age*	
18 -35 yo	37 (82.2%)
>35 yo	7 (15.6%)

*Ethnicity *	
Caucasian	3 (6.6%)
African American	42 (93.3%)

*Education Level*	
High School or less	23 (51.1%)
More than High school	22 (48.9%)

*Employment *	
Employed	13 (31.0%)
Unemployed	29 (69.1%)

*Age at HIV Infection*	
< 16 yo	7 (15.6%)
16 – 20 yo	13 (28.9%)
>/= 21 yo	25 (55.6%)

*Route of HIV Infection*	
Sexual Intercourse	28 (62.2%)
IV drug use	1 (2.2%)
Congenital	6 (13.3%)
Unknown/Chose not to disclose	10 (22.2%)

*Amount of time with HIV infection *	
</= 3 years	18 (40.9%)
4-10 years	14 (31.8%)
>10 years	12 (27.3%)

*Diagnosed during pregnancy*	
Yes	14 (33.3%)
No	28 (66.7%)

*Previously HIV infected children*	
Yes	1 (2.2%)
No	44 (97.8%)

*Gravida *	
1	5 (11.1%)
2	10 (22.2%)
>/=3	30 (66.7%)

*Previous pregnancy outcomes*	
Term	32 (71.1%)
Preterm	8 (17.8%)
Spontaneous Abortion	23 (51.1%)

**Table 2:** Medication adherence during pregnancy in cohort of HIV infected women (n=45).

*Medication adherence *	
Every day	35 (77.8%)
Most days	6 (13.3%)
Few days	2 (4.4%)
None	1 (2.2%)

*Reasons for missed doses*	
Forgot	25 (56.8%)
Could not afford	1 (2.3%)
Side effects	3 (6.8%)
Did not want to take	4 (4.6%)
Others	13 (29.6%)

**Table 3:** Knowledge of perinatal transmission among a cohort of HIV infected women (n=45).

	No	I don't know	Yes
HIV medications could harm my baby	36 (81.8%)	6 (13.6%)	2 (4.6%)

HIV medications will harm me	36 (83.7%)	5 (11.6%)	2 (4.7%)

Being pregnant will harm my health	36 (83.7%)	4 (9.3%)	3 (7.0%)

My child will be born HIV-positive even if I take my HIV medicines	35 (79.6%)	7 (15.9%)	2 (4.6%)

My child will likely not be born HIV-positive if I take my HIV medicines as prescribed in pregnancy	10 (22.7%)	4 (9.1%)	30 (68.2%)

Delivering vaginally puts my child at higher risk for becoming HIV-positive even if my viral load is undetectable	25 (58.1%)	10 (23.3%)	8 (18.6%)

Delivering by cesarean section may prevent my child from becoming HIV-positive	8 (18.6%)	9 (20.9%)	26 (60.5%)

Breast-feeding puts my child at risk for becoming HIV-positive	0	3 (7.0%)	40 (93.0%)

If I take my HIV medications regularly and have a viral load less than 1000, I can safely deliver vaginally and the risk of HIV transmission to my child would be <2%	3 (6.8%)	7 (15.9%)	34 (77.3%)

## Data Availability

The data is not readily available to the public. It can be requested from the authors. It is currently kept on a HIPAA compliant cloud based online application under password protection.

## Supplementary Materials

Supplementary Materials This supplementary file includes the survey utilized for the study.
