# Supplementary material for: Perceived Barriers to Antepartum HIV Medication Adherence in HIV Infected Pregnant Women
Source: Infect Dis Obstet Gynecol. 2018 Oct 16;2018:4049212. doi: 10.1155/2018/4049212 (PMC6206578; doi:10.1155/2018/4049212)
Supplement: Supplementary Materials — This supplementary file includes the survey utilized for the study. [file 4049212.f1.zip › 4049212.f1/4049212 supplementary files_IDOG_2502579.docx]

**SUPPLEMENTARY FILES**

**The provided survey was utilized for the study.**

**Survey**

Thank you for participating in this research study. We are trying to learn more about how to provide the best care to HIV positive women. We ultimately hope to find a way to prevent children of HIV positive women from becoming infected with HIV during pregnancy.

You will complete an online survey that will take approximately 10 -15 minutes. Your health care provider will also answer some questions about your medical history. Participation in this research is completely voluntary and you can stop at any time. Your responses will be linked to your medical record in order to follow the course of your newborn. Your name will not be utilized in any publications or presentations and all of our results will be coded and anonymous. By completing this survey, you are letting us know that you agree to participate in this study.

If you feel uncomfortable answering some of the questions, you do not have to answer them. Taking this survey will not benefit you directly, but we hope this research study will help us address how to better take care of pregnant HIV-infected women.

Your survey responses will be sent to a link on the survey website where data will be stored in a password protected electronic format. You will receive a participant number that will be entered into the survey and therefore no identifying information, such as your name, email address, or IP address, will be collected. No one will be able to identify you or your answers and no one will know whether or not you participated in the study. You will receive a small token valued at $5 upon completion of the survey as compensation.

If you ever have questions about this study you should contact Dr. Martina Badell at 404-933-6071. If you have questions about your rights as a research subject, you may contact the Emory University Institutional Review Board at (404) 712-0720 or toll free at 1-877-503-9797. If you have a question about your rights as a Grady patient, you may contact Dr. Curtis Lewis, Vice President of Medical Affairs, at (404) 616-4261.

**BACKGROUND**

1) How old are you?

❑ 18 – 20 years old

❑ 21 – 25 years old

❑ 26 – 30 years old

❑ 30 - 35 years old

❑ 35 – 40 years old

❑ > 40 years old

2) Are you Hispanic or Latina? ☐YES ☐NO

3) What is your racial background? *Check all that apply*

☐ White ☐ Black or African American
☐ Asian ☐ Native Hawaiian or Pacific Islander
☐ American Indian or Alaska Native ☐ Other ______________________

4) Check the highest level of education you have COMPLETED.

❑ Elementary school ❑ Some college or technical school

❑ High school ❑ College

❑ Graduate school (J.D./Ph.D./M.D./Master’s degree)

5) What is your current employed status?

❑ Employed

❑ Unemployed due to medical illness

❑ Unemployed due to another reason

❑ Can’t find work

❑Other: ____

6) What is your monthly income?

❑ <$1000/mo

❑ $1000 - $2000/mo

❑ > $2000/mo

❑ Choose not to say

7) Who do you live with currently? Check all that apply

❑ Partner/spouse/significant other

❑ Your children

❑ Other family members

❑ Alone

❑ In shelter

❑ Homeless

8) How long have you known you were HIV positive?

❑ Found out during this pregnancy

❑ 1 – 3 years

❑ 4 – 10 years

❑ > 10 years

9) How old were you when you were told you had HIV?

❑ <10 years old

❑ 10 – 15 years old

❑ 16 – 20 years old

❑ 21 – 25 years old

❑ 26 – 30 years old

❑ > 30 years old

10) How did you get HIV?

❑ Sex with a man/men

❑ Intravenous drug use

❑ Congenital – from your mom

❑ Choose not to say

❑ Unsure

❑ Other

***For questions 11-14, please include your current pregnancy in the number of pregnancies you report.***

11) How many times have you EVER been pregnant (including abortions or miscarriages)? *Please circle.*

1 2 3 4 5 6 7 8 9 Other ______

12) How many times have been pregnant SINCE you were diagnosed with HIV (including abortions or miscarriages)? *Please circle.*

1 2 3 4 5 6 7 8 9 Other ______

13) How many times have you given birth EVER in your lifetime? *Please circle.*

1 2 3 4 5 6 7 8 9 Other ______

14) How many times have you given birth SINCE you were diagnosed with HIV? *Please circle.*

1 2 3 4 5 6 7 8 9 Other ______

15) Have any of your children been found to be HIV positive after delivery?

☐ Yes ☐ No

16) Please check the ONE statement which most applies to you:

☐ I found out I had HIV during this current pregnancy.

☐ I found out I had HIV during a past pregnancy.

☐ I found out I had HIV at a time when I was not pregnant.

17) Are you able to identify the father of your current pregnancy?

☐YES ☐NO, *SKIP to question number 18*

17a) Does the father of your current pregnancy have HIV? ☐YES ☐NO ☐DON’T KNOW

17b) Does the father of your current pregnancy know about your HIV infection?

☐YES ☐NO ☐DON’T KNOW

**CIRCUMSTANCES OF PREGNANCY**

Below are some questions that ask about your life and feelings around the time you became pregnant this time. Please think of your current pregnancy when answering the questions below.

18) Did you utilize drugs, alcohol, or cigarettes prior to your pregnancy?

☐ Yes ☐No

19) If you were utilizing drugs, alcohol, or cigarettes prior to your pregnancy did you continue utilizing them during your pregnancy?

☐ Yes ☐No

20) Just before I became pregnant this time....

***(Please check the ONE statement which most applies to you)***

☐ I wanted to have a baby

☐ I had mixed feelings about having a baby

☐ I did not want to have a baby

21) Before you became pregnant, did you do anything in preparation for pregnancy?

***(Please check ALL that apply)***

☐ took folic acid or prenatal vitamins

☐ took my HIV medicines (anti-retrovirals) every day

☐ stopped or cut down drinking alcohol or using drugs or smoking cigarettes

☐ ate more healthily

☐ got medical/health advice

☐ did something else to prepare, please describe ____________________________

or

☐ I did not do any of the above before my pregnancy

**22) What do you think the chances are that your baby will be born with HIV? (Please give a percentage anything between 0-100%)**

**______**

23) *The next questions ask about your understanding of the use of HIV medicines in pregnancy.*

**Please tell answer yes, no, or I don’t know to the following questions***: (Circle your choice.)*

|  | No | I don’t know | Yes |
| --- | --- | --- | --- |
| HIV medications could harm my baby. | 1 | 2 | 3 |
| HIV medications will harm me. | 1 | 2 | 3 |
| Being pregnant will harm my health. | 1 | 2 | 3 |
| My child will be born HIV-positive even if I take my HIV medicines. | 1 | 2 | 3 |
| My child will likely not be born HIV-positive if I take my HIV medicines as prescribed in pregnancy. | 1 | 2 | 3 |
| Delivering vaginally puts my child at higher risk for becoming HIV-positive even if my viral load is undetectable. | 1 | 2 | 3 |
| Delivering by cesarean section may prevent my child from becoming HIV-positive. | 1 | 2 | 3 |
| Breast-feeding puts my child at risk for becoming HIV-positive. | 1 | 2 | 3 |
| If I take my HIV medications regularly and have a viral load less than 1000, I can safely deliver vaginally and the risk of HIV transmission to my child would be <2%. | 1 | 2 | 3 |

**HEALTHCARE DURING PREGNANCY**

24) Did you get routine and scheduled prenatal care this pregnancy?

☐YES ☐NO

25) Did a doctor, nurse, or social worker explain that HIV medicines (antiretrovirals) could lower the chance that you pass HIV to a baby during pregnancy?

☐YES ☐NO

26) If you had to miss an appointment what was the reason?

☐ No ride, transportation issues

☐ Could not afford

☐ I did not have time

☐ I did not have anyone to take care of my other children

☐ Other reason: ________________

27) Were you on HIV medicines (antiretroviral medication) prior to this pregnancy?

☐ Yes ☐ No

28) Were you advised by a doctor, nurse, or social worker to take HIV medicines this pregnancy?

☐ Yes ☐ No

29) If yes, during an average week what percentage of the time did you take the medication?

☐ Every day

☐ Most days

☐ Some days

☐ Few days

☐ None

30) If you missed doses of your medication, what was the reason?

☐ I forgot

☐ I could not afford the medicine

☐ Bad side effects

☐ I did not want to take the medicine

☐ Other:________

31) If there was a time during your pregnancy you stopped taking your medication, why did you do so?

❑ Nausea and vomiting

❑ Lack of insurance

❑Concern for baby

❑ Could not afford

❑ Other:________

32. What did you use to help you remember to take your medications everyday?

❑ Set an alarm on phone

❑ Placed medication bottle in same place as reminder every day

❑ Other: __

❑ I didn’t use anything to help me remember

33) In your opinion, which of the medical providers you saw throughout pregnancy was most helpful in assisting and discussing taking HIV medications and coming to appointments?

❑ Nurses

❑ Social worker

❑ Doctor

❑ Other: _________

34) Did you talk with other HIV+ moms during your pregnancy for advice and support?

❑ Yes

❑ No

35) Do you think a scheduled support group meeting with other HIV positive moms would have helped you with taking your medications during pregnancy?

❑ Yes

❑ No

36) Was your partner supportive of you taking your medications?

❑ Yes

❑ No

❑ No partner involved

37) Can you think of anything that could have been done to make taking your HIV medications during pregnancy easier for you?

**THANK YOU FOR YOUR TIME.**
